# Bionic hand exoprosthesis – Perspectives for the future in Romania

**Published:** 2014

**Authors:** ME Pogarasteanu, AG Barbilian

**Affiliations:** * Orthopaedics–Traumathology Clinic, “Dr. Carol Davila” Central Military University Emergency Hospital, Bucharest, Romania

**Keywords:** bionic hand, circumferential osteoneuromioplasty, myoelectric signals

## Abstract

Prosthetics is a modern area of interest and a challenge in Orthopedics. Over time, there has been a transition from an artisanal method of prosthetics production to modern concepts and materials, including a re-education through virtual reality. The conditions for an efficient fitting of a prosthesis include the necessity that the prosthesis respects the form and function of the lost limb, both anatomically and biomechanically. Prosthetics are made individually, personalized according to sex, age, physiological characteristics, profession and preference. In our country, thoracic limb prosthetics has a relatively short-recorded history of approximately a century, the most preeminent centers being in Iasi, Cluj and Bucharest. Currently, thoracic limb prosthetics, and particularly hand prosthetics, are in a period of development. A technique for amputation and stump reamputation called “circumferential osteoneuromioplasty” (CONM) is currently being used in the Orthopedics and Trauma Clinic of the Central Military University Emergency Hospital in Bucharest. The method was created with the purpose of obtaining distinct myoelectric signals of better quality, following the contraction of each muscle. The CONM method can be used in conjuncture with both the new hand prostheses that are currently available in Romania, and with the model that is being developed by a mixed team from the Polytechnic University in Bucharest, in collaboration with the Central Military University Emergency Hospital in Bucharest.

## Introduction

Prosthetics is a modern area of interest and a challenge in Orthopedics, as the loss of a limb and its replacement with an artificial one is still regarded as a medical failure.

Essentially, the act of amputation in all the cases is accompanied by a certain degree of psychological trauma; for this reason, the patient reeducation is a three-step process: pre-amputation, post-amputation in the hospital, and post-amputation with a re-adaptation after the release from hospital care. This last stage includes the actual fitting of a prosthesis, initially with a temporary prosthesis, useful in stabilizing the characteristics of the amputation stump (stump maturation), then with a final prosthesis. In order to ensure the success of this stage, an amputation stump that has a healthy skin, is well vascularized, has a good sensibility, lacks pain, and presents no adherences, is needed. The scar tissue must have a normal aspect, without excessive sensibility or adherences to the underlying structures. The musculature must be balanced according to the antagonist groups; this is optimally accomplished through the procedure known as osteomyoplasty.

Over time, there has been a transition from the artisanal method of prosthetics production to modern materials and concepts, including the re-education through the virtual reality and the 3D printing of the prosthetic components. Progresses in this area have allowed patients to return to a normal lifestyle, both professionally and personally, with an acceptable degree of satisfaction. Recent innovations allow the constructing of upper limb prosthetics specifically designed for an activity, such as fishing or baseball.

The conditions for an efficient fitting of a prosthesis include the necessity that the prosthetic respects the shape and the function of the lost limb, both anatomically and biomechanically; the hardest to restore is the function, although there is a large variety of modern materials and techniques. Also, the prosthesis needs to respect the original degree of sensibility as much as possible, allowing an ample degree of feed-back with the environment.

Regarding the upper limb, the objectives of a prosthetic device may be summed up as it follows: to obtain the greatest degree of mobility, to accomplish prehension, and to adapt to what is left of the stump’s sensibility and anatomy. The more proximal the amputation site is, the harder these objectives are attained.

During the process of production, each prosthesis is individualized, adapted to the patient, so that it is symmetric to the other limb. We may define the upper arm prosthesis as external medical devices, with the purpose of anatomically, functionally and aesthetically replace the lost limb, either wholly or partially. The prosthesis is individual, personalized according to sex, age, physiological characteristics, profession and preferences.

Still, it is accepted that a prosthesis that can completely replace the lost anatomic segment has not been developed yet, this goal remaining an open challenge.

**The history of upper limb prosthetics in Romania**

The upper limb prosthetics has had a relatively short recorded history, of approximately a century, in Romania.

The orthopedic surgeon Ghiulamila ran the only prosthetics workshop in Romania in the years before the First World War. Between 1916 and 1918, the first officially recognized prosthetics workshop was opened in Iasi, with the goal of ensuring prosthetics for the soldiers and civilians wounded in the First World War.

In 1921, Doctor Al. Radulescu began running the activities of the Institute for Orthopedics in Cluj. He will be the one to take over the prosthetics workshop in Cluj, remaining as an annex after the Institute became the Orthopedics Hospital.

Almost two decades later, in 1940, the workshop was moved to Bucharest, with the qualified personnel who worked there; in the capital city, the workshop saw an increase in activity and personnel (up to 125 skilled workers), with the opening of a new section [**1**].

The current day status of the upper body prosthetics in Romania was that of a field in development, with a few notable successes and great possibility for innovation and originality.

**Perspectives for the future: The CONM technique**

There is a concept in current-day prosthetics that the new methods and materials used in the field must be matched with, by new surgical methods of amputation and stump revision, in order to amplify each other’s efficiency. The aim is to develop an innovative surgical method, having the final end objective of providing the patient with a bionic exoprosthesis, fitted with a higher number of sensors.

Currently, a technique for amputation and stump reamputation called “circumferential osteoneuromioplasty” (CONM) is being used in the Orthopedics-Trauma Clinic of Central Military University Emergency Hospital in Bucharest. The technique has been conceived by Prof. Adrian Barbilian, MD, from “Carol Davila” University of Medicine and Pharmacy, Bucharest, Romania.

The method was designed to obtain distinct myoelectric signals of enhanced clarity, following the contraction of each muscle individually. CONM technique is used in the Clinic in the upper arm amputation and in stump revisions preceding the fitting of a prosthesis. The aim is to decrease the distance between the deeper, muscle layer and the skin, and also to individualize each muscle, in order to collect better EMG signals, with superior clarity, by sensors fitted on the myoprosthesis.

**Fig. 1:**
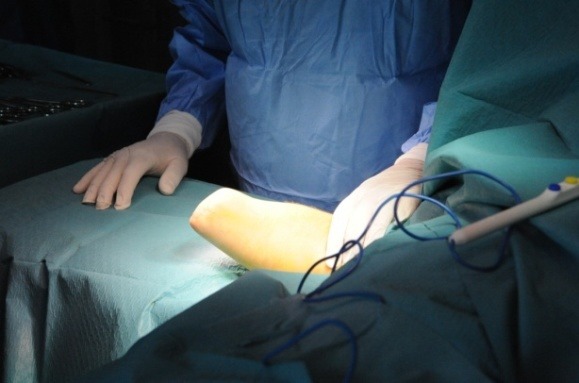
Forearm stump

The CONM technique includes the freeing, identification and repositioning of muscles, while suturing them according to muscle groups and motor functions, aiming to collect as many EMG signals as possible, that can then be used by a prosthesis with multiple functions, thus coming close to a natural movement of the partially amputated upper limb.

**Fig. 2:**
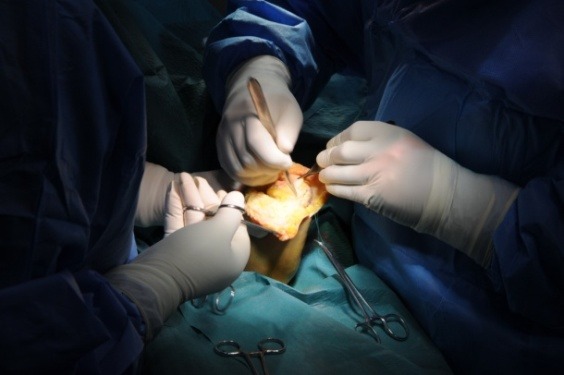
Superficial dissection with fasciotomy in a forearm stump revision by using the CONM technique

Each muscle is identified and then reinserted circumferentially, anchoring them through the bone, thus preventing their retraction, while respecting their anatomical disposition in groups.

**Fig. 3:**
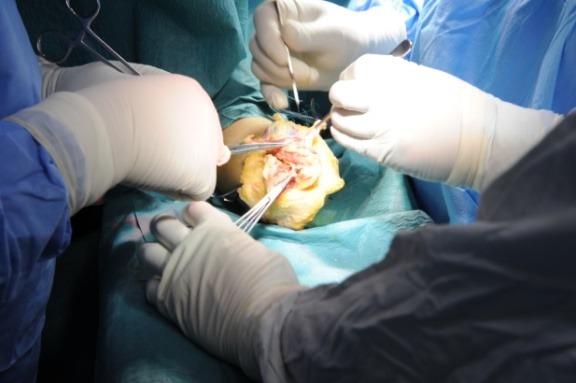
Dissection is carried out in order to identify the muscle groups in a forearm stump revision

Also, the CONM technique involves fasciotomy at the level of the amputation stump, in order to reduce the electric resistance between muscle and skin, also involving the surgical freeing of the nerves from the fibrotic scar tissues surrounding them, in order to bring the nerves closer to the surface, for a better reception of EMG signals [**2**,**3**].

**Fig. 4:**
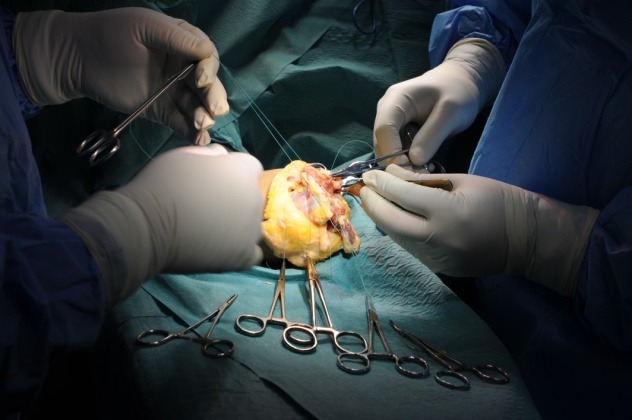
The muscle groups are isolated and positioned for the CONM procedure in a forearm stump revision

The CONM technique may be applied in conjuncture with the new hand prosthesis available in Romania and with the model that is being developed in the Polytechnic University in Bucharest, by a mixed team, in collaboration with the “Dr. Carol Davila” Central Military University Emergency Hospital in Bucharest. The new type of bionic hand will be equipped with a greater number of functions, in great part thanks to the application of the CONM technique, which has allowed the reception of a greater number of distinct and clear EMG signals; the patient will be trained by using virtual reality, through an innovative technique that uses biofeedback [**4**,**5**].

The future of upper limb prosthetics lies in the author’s vision, in joining concepts between surgeons and prosthetics’ manufacturers and fitters, having the patient’s benefit at heart.

**Acknowledgements**

This work was supported by the staff in the Orthopaedics–Traumathology Clinic in the Central Military University Emergency Hospital in Bucharest, to whom we would like to extend our gratitude.
